# Global multicenter validation of noninvasive fibrosis assessment pathways in MetALD and ALD

**DOI:** 10.1097/HC9.0000000000001020

**Published:** 2026-07-27

**Authors:** Hyundam Gu, Luis Antonio Díaz, Natalia Baeza, Francisco Idalsoaga, Xiao-Dong Zhou, Ming-Hua Zheng, Rakhi Maiwall, Shiv K. Sarin, Anand V. Kulkarni, Ramagundam Ramyasri, Fátima Higuera de la Tijera, Helena Cortez Pinto, Sofia Carvalhana, Rita Fernandes, Mohammad Qasim Khan, William Alazawi, Wenhao Li, Laura Temperley, David Marti-Aguado, Jordi Gratacós-Ginès, Elisa Pose, Maria Poca, Berta Cuyas, Pedro Montes, Fernando Javier Barreyro, Gustavo Ayares, Terry Cheuk-Fung Yip, Vincent Wai-Sun Wong, Grace Lai-Hung Wong, Jimmy Che-To Lai, Cristiane Villela-Nogueira, Nathalie Leite, Gil Fernando Salles, Claudia Regina Lopes Cardoso, Daniza Contreras, José Antonio Velarde-Ruiz Velasco, Alceo Galimberti, Fernando Bessone, Francisco Javier Valentin-Cortez, Alejandra Mijangos-Trejo, Norberto C. Chavez-Tapia, Ezequiel Ridruejo, Mirta Peralta, Juan Pablo Roblero, Daniela Simian, Pamela Gil, Mohamed El-Kassas, Angelo Z. Mattos, Johana Acuña, Roberta Chaves Araujo, Igor Lima Ferraz, Katherine Marrugo, Alonso Vera Torres, Juan Diego Torres, Valentina Mejía Valdés, Jacqueline Córdova, Manuel Mendizabal, Claudia Alves Couto, Guilherme Grossi Lopes Cançado, Thomas G. Cotter, Ahmad Anouti, Elias D. Rady, Hamza Dahshi, Sheel Patel, Ashwani K. Singal, Katrina Pekarska, Richard Parker, Mohamad Ali Ibrahim, Prasun K. Jalal, Graciela Castro-Narro, Mazen Noureddin, Naim Alkhouri, Winston Dunn, Patrick S. Kamath, Arun Sanyal, Richard K. Sterling, Veeral Ajmera, Rohit Loomba, Marco Arrese, Ramón Bataller, Juan Pablo Arab

**Affiliations:** 1 Department of Internal Medicine, Division of Gastroenterology, Hepatology, and Nutrition, Virginia Commonwealth University School of Medicine, Richmond, Virginia, USA; 2 MASLD Research Center, Division of Gastroenterology and Hepatology, University of California San Diego, San Diego, California, USA; 3 Departamento de Gastroenterología, Escuela de Medicina, Pontificia Universidad Católica de Chile, Santiago, Chile; 4 Department of Medicine, Division of Gastroenterology, Western University, London Health Sciences Center, London, Ontario, Canada; 5 MAFLD Research Center, Department of Hepatology, The First Affiliated Hospital of Wenzhou Medical University, Wenzhou, Zhejiang Province, China; 6 Department of Hepatology, Institute of Liver and Biliary Sciences, Delhi, India; 7 Department of Hepatology and Liver Transplantation, AIG Hospitals, Hyderabad, India; 8 Departamento de Gastroenterología, Hospital General de México “Dr. Eduardo Liceaga”, Ciudad de México, Mexico; 9 Serviço de Gastrenterologia e Hepatologia, Unidade Local de Saúde de Santa Maria, Lisbon, Portugal; 10 Faculdade de Medicina, Universidade de Lisboa, Lisbon, Portugal; 11 Barts Liver Centre, Blizard Institute, Queen Mary University of London, London, UK; 12 Digestive Disease Department, Clinic University Hospital, INCLIVA Health Research Institute, Valencia, Spain; 13 Liver Unit, Hospital Clínic de Barcelona, Institut d’Investigacions Biomèdiques Augusut Pi i Sunyer (IDIBAPS), Barcelona, Spain; 14 Department of Gastroenterology, Hospital de la Santa Creu i Sant Pau, Institut de Recerca Hospital de Sant Pau-IIB Sant Pau, Universitat Autònoma de Barcelona, CIBERehd, Barcelona, Spain; 15 Departamento de Gastroenterología, Hospital Nacional Daniel Alcides Carrión, Bellavista, Peru; 16 Departamento de Gastroenterología, Escuela de Medicina, Universidad Nacional de Misiones, Misiones, Argentina; 17 Medical Data Analytics Center, Department of Medicine and Therapeutics, The Chinese University of Hong Kong, Hong Kong SAR, China; 18 División de Hepatología, Escola de Medicina, Hospital Universitário Clementino Fraga Filho, Universidade Federal do Rio de Janeiro, Rio de Janeiro, Brazil; 19 Instituto Nacional de Diabetes, Endocrinología y Nutrición (INDEN), Santo Domingo, Dominican Republic; 20 Hospital Civil de Guadalajara Fray Antonio Alcalde, Guadalajara, Mexico; 21 Departamento de Clínicas Medicas, CUCS, Universidad de Guadalajara, Guadalajara, Mexico; 22 Department of Gastroenterology, Hospital Centenario de Rosario, Santa Fe, Argentina; 23 Department of Gastroenterology and Obesity, Medica Sur Hospital, Mexico City, Mexico; 24 Hepatology Section, Department of Medicine, Centro de Educación Médica e Investigaciones Clínicas Norberto Quirno “CEMIC”, Buenos Aires, Argentina; 25 Hospital Francisco J. Muñiz, Ciudad Autónoma de Buenos Aires, Argentina; 26 Sección de Gastroenterología, Departamento de Medicina Interna, Hospital Clínico Universidad de Chile, Santiago, Chile; 27 Endemic Medicine Department, Faculty of Medicine, Helwan University, Cairo, Egypt; 28 Gastroenterology and Hepatology Unit, Irmandade Santa Casa de Misericórdia de Porto Alegre, Porto Alegre, Rio Grande do Sul, Brazil; 29 Divisão de Gastroenterologia do Hospital das Clínicas da Faculdade de Medicina de Ribeirão Preto - Universidade de São Paulo, Ribeirão Preto, SP, Brasil; 30 Department of Hepatobiliary Surgery and Transplants, Hospital Universitario Fundación Santa Fe de Bogotá, Bogotá, Colombia; 31 Hospital General Manuel Gea González, Mexico City, Mexico; 32 Unidad de Hígado y Trasplante Hepático, Hospital Universitario Austral, Pilar, Argentina; 33 Instituto Alfa de Gastroenterologia, Hospital das Clínicas da Universidade Federal de Minas Gerais, Belo Horizonte, MG, Brazil; 34 Division of Digestive and Liver Diseases, UT Southwestern Medical Center, Dallas, Texas, USA; 35 Evidentis Clinical Research, Fort Worth, Texas, USA; 36 Department of Pediatrics, UT Southwestern Medical Center, Dallas, Texas, USA; 37 Division of Gastroenterology, Hepatology and Nutrition, University of Louisville School of Medicine, Louisville, Kentucky, USA; 38 Leeds Liver Unit, Leeds Teaching Hospitals NHS Trust, Leeds, UK; 39 Department of Gastroenterology and Hepatology, Baylor College of Medicine, Houston, Texas, USA; 40 Department of Gastroenterology, Instituto Nacional de Ciencias Médicas y Nutrición “Salvador Zubirán”, Mexico City, Mexico; 41 Houston Methodist Hospital, Houston, Texas, USA; 42 Department of Hepatology, Summit Clinical Research, San Antonio, Texas, USA; 43 University of Kansas Medical Center, Kansas City, Kansas, USA; 44 Division of Gastroenterology and Hepatology, Mayo Clinic, Rochester, Minnesota, USA

**Keywords:** alcohol-associated liver disease, cirrhosis, hepatology, metabolic dysfunction and alcohol-associated liver disease, steatotic liver disease

## Abstract

**Background:**

Noninvasive tests are well validated in metabolic dysfunction–associated steatotic liver disease, but evidence in metabolic dysfunction and alcohol-associated liver disease (MetALD) and alcohol-associated liver disease (ALD) is limited. We evaluated noninvasive test–based clinical care pathways for fibrosis risk stratification in MetALD and ALD.

**Methods:**

This is a multinational, multicenter, cross-sectional study across 35 centers in 16 countries (2013–2025), including adults with MetALD or ALD. Fibrosis was assessed by liver biopsy and/or vibration-controlled transient elastography (VCTE). In biopsy-proven participants, we analyzed the sequential pathway (Fibrosis-4 [FIB-4] first, followed by VCTE for indeterminate FIB-4) to identify advanced fibrosis (≥F3). Diagnostic accuracy was assessed using the AUC.

**Results:**

Among 893 participants (41.3% MetALD and 58.7% ALD), the median age was 52 [43.0–61.0] years, and 79.4% were male. The estimated prevalence of advanced fibrosis was 45% (32.2% in MetALD and 54.0% in ALD). Participants with MetALD had a more dysmetabolic profile but lower fibrosis stages than ALD. Among biopsy-proven participants, FIB-4 AUC was 0.720, and VCTE AUC was 0.833. FIB-4 performed better in MetALD than ALD (AUC 0.773 vs. 0.540), while VCTE accuracy was similar across groups. In the sequential pathways, the false-negative rate for advanced fibrosis (misclassified as low risk) was 6.8% (4.9% MetALD and 11.3% ALD).

**Conclusions:**

In biopsy-proven patients, FIB-4 performance was acceptable in MetALD but substantially weaker in ALD. The standard 2-step pathway performed adequately in MetALD but missed a clinically meaningful proportion of advanced fibrosis in ALD.

## Graphical abstract

**Figure:**
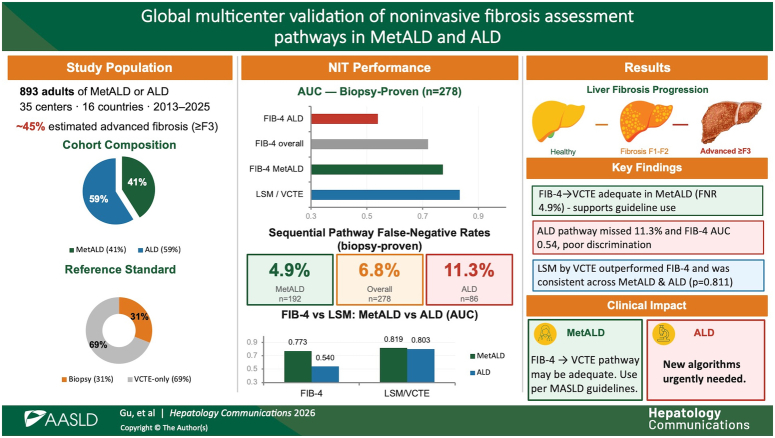

## INTRODUCTION

Metabolic dysfunction–associated steatotic liver disease (MASLD) and alcohol-associated liver disease (ALD) are now classified within the broader category of steatotic liver disease (SLD), a unifying nomenclature that encompasses chronic liver disease characterized by pathological hepatic fat accumulation.^1^ This definition also recognizes the overlap between MASLD and ALD, termed metabolic dysfunction and alcohol-associated liver disease (MetALD).^2^ This entity is diagnosed by the presence of metabolic dysfunction and alcohol use between 140 and 350 g/wk in women and between 210 and 420 g/wk in men.^3^ MetALD has important therapeutic implications in clinical practice, as it is associated with an accelerated progression to advanced stages of liver disease compared with MASLD.^4^^,^^5^

Advanced liver fibrosis, defined as fibrosis stage ≥3, including cirrhosis, is now recognized as a key determinant of liver-related outcomes in SLD, consistent with contemporary consensus recognizing advanced fibrosis as a clinically meaningful risk threshold.^6^^–^^8^ For many years, liver biopsy has been considered the gold standard for staging liver fibrosis. Given the well-recognized limitations of liver biopsy,^9^ noninvasive tests (NITs) have largely superseded biopsy for fibrosis-based risk stratification.^10^ Stepwise risk stratification via NIT-based clinical care pathways is the standard of care, with the Fibrosis-4 (FIB-4) index used for initial triage and liver stiffness measurement (LSM) by vibration-controlled transient elastography (VCTE) as second-line testing in individuals at intermediate risk.^11^^–^^14^ This strategy seeks to identify patients with fibrosis who are most likely to benefit from hepatology referral or therapeutic intervention, while minimizing unnecessary evaluations for low-risk individuals.

The diagnostic performance of these NITs is relatively well characterized in MASLD, partly because the tools and pathways were developed and validated predominantly in MASLD. In contrast, evidence in ALD, and particularly in MetALD, remains limited.^10^ Alcohol, as an additional cofactor, introduces additional biological and analytic complexity that may influence NIT performance, including effects on aminotransferases and LSM, independent of fibrosis. Moreover, most available evidence in MetALD has evaluated the individual performance of NITs and consists primarily of single-center studies. Therefore, this multinational, multicenter, cross-sectional study aims to assess the performance of FIB-4 and VCTE within clinical care pathways among participants with MetALD and ALD for stratifying the risk of liver fibrosis.

## METHODS

### Study design and participants

This cross-sectional study assessed the performance of care pathways in individuals with MetALD and ALD according to the 2023 SLD criteria.^15^ This study was conducted at 35 centers in 16 countries between 2013 and 2025 (Supplemental Table S1, https://links.lww.com/HC9/C442). We recorded clinical history, imaging, laboratory investigations, and liver biopsy data where available. We collected the FIB-4 test and LSM data from VCTE in all participants for whom data were available. This study was approved by the institutional review board at each participating center. Due to its retrospective nature, informed consent was waived at each participating center. This study was conducted under Good Clinical Practice guidelines, the Declaration of Helsinki and Istanbul, and adheres to the Standards for Reporting of Diagnostic Accuracy (STARD) guidelines.^16^ The authors had access to the data and participated in analyses and drafting the manuscript.

**FIGURE 1:**
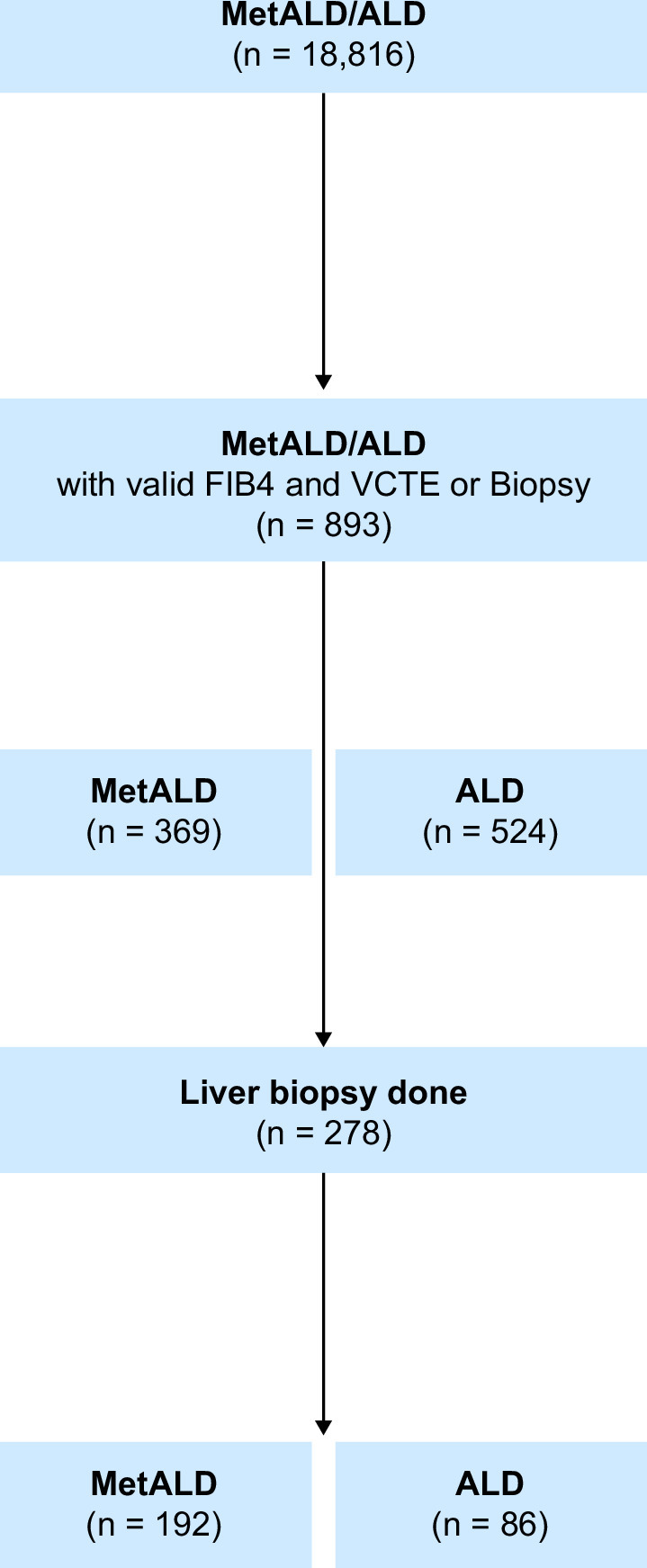
Flowchart diagram for the study population. Abbreviations: ALD, alcohol-associated liver disease; MetALD, metabolic dysfunction and alcohol-associated liver disease.

Participants who met the following criteria were included in the study: (1) age ≥18 years, (2) MetALD or ALD diagnosis according to the 2023 SLD criteria, and (3) assessment of liver fibrosis by using liver biopsy and/or VCTE. We excluded participants with laboratory evidence of liver disease other than MetALD or ALD. The Consolidated Standards of Reporting Trials (CONSORT) flowchart is shown in Figure 1.

### Clinical assessment and laboratory tests

We registered the basic sociodemographic and clinical data. Individuals were categorized by self-reported race/ethnicity. Hispanics were defined as individuals from Latin America with Spanish-speaking origin, regardless of race. Participants from Brazil (a Portuguese-speaking country) were categorized by race, such as non-Hispanic White or non-Hispanic Black. Alcohol intake was assessed by self-report and recorded as grams per week. Participants were specifically asked about the frequency, quantity, and type of alcoholic beverages consumed, and the presence of binge drinking, using validated patient questionnaires and standardized clinical interviews at each participating center. Objective biomarkers of alcohol use, such as phosphatidylethanol (Peth), were not routinely available across participating centers during the study period. We collected the following biochemical tests: AST, ALT, total cholesterol, triglycerides, HDL-cholesterol, LDL-cholesterol, ALP, total bilirubin, albumin, creatinine, γ-GT, and platelets.

### VCTE assessment and liver biopsy

LSM by VCTE was obtained using FibroScan (Echosens). An experienced technician performed VCTE after a 4-hour minimum fast. We performed at least 10 repeated valid measurements during patient breath-holding (assessed automatically by the FibroScan system). All participants were first scanned using the M probe (3.5 MHz). If indicated upon initial assessment, participants were re-scanned using the XL probe (2.5 MHz). In some centers, VCTE with an automatic probe selection tool was used. All VCTE examinations were performed by trained operators following a standardized protocol across participating centers, and study coordinators underwent formal training and certification in FibroScan operation before study participation.

Liver biopsies were performed in each center according to local regulations and protocols. Liver biopsies were graded and staged by a local pathologist(s) using the nonalcoholic steatohepatitis Clinical Research Network histologic scoring system, scoring fibrosis from 0 to 4. We only included clinical data obtained within 6 months of LSM by VCTE or liver biopsy.^17^ In evaluation, if a liver biopsy was not available, we included a VCTE performed within 6 months, as an explanatory analysis to increase the number of valid observations and improve generalizability. Steatosis was defined using liver biopsy when available (>5% of hepatocytes), or by abdominal ultrasound demonstrating diffuse hepatic hyperechogenicity, a controlled attenuation parameter (CAP) ≥275 dB/m, or magnetic resonance imaging–proton density fat fraction ≥5% (if liver biopsy was not available).^18^

### Noninvasive blood tests and clinical care pathways

The FIB-4 index was calculated using the original formula, with FIB-4 values of <1.3 classified as low risk, 1.3–2.67 as indeterminate risk (2.0–2.67 for individuals aged over 65 y), and ≥2.67 as high risk in accordance with the clinical care pathways, as these thresholds can identify liver fibrosis and predict all-cause and liver-related outcomes.^11^^–^^14^ Although the FIB-4 index may have a lower accuracy than other serum-based fibrosis markers, it is recommended due to its wide availability, simplicity, and low cost.^12^ In line with these recommendations, LSM by VCTE was categorized as <8 kPa (low risk), 8–12 kPa (indeterminate risk), and ≥12 kPa (high risk) for second-line fibrosis risk stratification.^11^^–^^14^

### Primary and secondary endpoints

The primary endpoint was the performance of clinical care pathway algorithms, specifically the sequential application of FIB-4 followed by VCTE in participants with a liver biopsy available. The secondary outcomes included the individual diagnostic performance characteristics of FIB-4 to detect advanced fibrosis both in the biopsy-proven and VCTE-only cohorts (as an explanatory analysis), and the differences in clinical characteristics among all included participants with MetALD and ALD.

### Statistical analysis

Continuous variables were summarized as mean and SD or as median with IQR, depending on the distribution. Comparisons between groups were performed using the Student *t* test or the Wilcoxon rank-sum test for continuous variables and the chi-test or Fisher exact test for categorical variables. For clinical pathways, diagnostic categories were classified as low risk, indeterminate risk, or high risk according to the aforementioned cutoffs recommended in clinical guidelines. The false-negative rate for clinical pathways was estimated as the proportion of cases with advanced fibrosis that were not identified using first- and second-line testing. The individual diagnostic accuracy of NITs was evaluated using the AUC with 95% CIs. AUCs were calculated for FIB-4 and LSM using VCTE to assess their ability to detect advanced fibrosis, defined as histologic fibrosis stage ≥3. For FIB-4, when liver biopsies were not available, performance for detecting advanced fibrosis was also evaluated in individuals with a LSM by VCTE ≥12 kPa. Pairwise comparisons of AUCs were conducted using DeLong’s method for independent receiver operating characteristic curves. Sensitivity, specificity, positive predictive value (PPV), and negative predictive value (NPV) were reported at prespecified thresholds. A 2-sided *p* value <0.05 was considered statistically significant. All analyses were conducted using R statistical software (R Foundation for Statistical Computing).

## RESULTS

### Characteristics of the study population

A total of 893 participants who had a valid FIB-4 value and LSM or biopsy results were identified (Figure 1). Of them, 369 (41.3%) individuals had MetALD and 524 (58.7%) had ALD. A total of 278 (31.1%) participants had a liver biopsy, and 615 (68.9%) underwent VCTE only. The estimated prevalence of advanced fibrosis within the study cohort was 45% (either biopsy-proven or by VCTE if biopsy was missing). The median age was 52.0 [43.0–61.0] years, and 79.4% were male. Race and ethnicity comprised 29.0% non-Hispanic White, 0.4% non-Hispanic Black/African American, 31.8% Hispanic, 37.1% Asian, and 1.7% of undisclosed or other races or ethnicities. The median BMI was 28.0 [24.5–31.6] kg/m^2^, while 27.2% had type 2 diabetes, 43.7% had hypertension, and 46.9% had dyslipidemia. The median FIB-4 index was 1.8 [1.0–3.5] and median LSM was 9.6 [6.1–26.6] kPa. By category, 39.1% had a low FIB-4, 26.5% had an indeterminate FIB-4, and 34.4% had a high-risk FIB-4; and 40.4% had LSM <8 kPa, 16.5% had LSM 8–12 kPa, and 43.1% ≥12 kPa.

Participants with MetALD were slightly older (53.0 vs. 51.0 y, *p*=0.006) with higher BMI (28.4 vs. 27.8 kg/m^2^, *p*=0.013). MetALD had a more dysmetabolic profile with higher rates of type 2 diabetes (31.2% vs. 24.2%, *p*=0.022) and dyslipidemia (61.7% vs. 36.5%, *p*<0.001). LSM by VCTE was lower in MetALD compared with ALD (median LSM 8.1 vs. 13.5 kPa; *p*<0.001), consistent with a lower prevalence of estimated advanced fibrosis (28.8% vs. 52.6%; *p*<0.001); FIB-4 categories also favored lower risk (FIB-4 <1.3: 47.7% vs. 33.0%, whereas FIB-4 ≥2.67: 20.3% vs. 44.3%; *p*<0.001). Advanced fibrosis was also less frequent in MetALD than ALD (32.2% vs. 54.0%; *p*<0.001) (Table 1).

**TABLE 1:** Baseline characteristics of participants with steatotic liver disease in the overall cohort

Characteristics	Global (N=893)	MetALD (N=369)	ALD (N=524)	*p* ^a^
Age (y)	52.0 [43.0–61.0]	53.0 [44.0–62.0]	51.0 [42.0–60.0]	0.006
Male, n (%)	709 (79.4)	299 (81.0)	410 (78.2)	0.353
Race/ethnicity, n (%)				<0.0001
NH White	259 (29.0)	149 (40.4)	110 (21.0)	
NH Black/ African American	4 (0.4)	0 (0.0)	4 (0.8)	
Hispanic	284 (31.8)	76 (20.6)	208 (39.8)	
Asian	331 (37.1)	141 (38.2)	190 (36.3)	
Undisclosed or other	14 (1.7)	3 (0.8)	11 (2.1)	
Body mass index (kg/m^2^)	28.0 [24.5–31.6]	28.4 [25.3–31.8]	27.8 [24.1–31.1]	0.013
Weight (kg)	78.2 [68.2–90.0]	80.0 [71.0–93.0]	77.0 [67.0–89.0]	<0.0001
Type 2 diabetes mellitus, n (%)	242 (27.2)	115 (31.4)	127 (24.2)	0.022
Hypertension, n (%)	389 (43.7)	161 (44.0)	228 (43.5)	0.942
Dyslipidemia, n (%)	417 (46.9)	226 (61.7)	191 (36.5)	<0.0001
Laboratory testing				
AST (IU/mL)	40.0 [27.0–65.0]	37.0 [26.0–58.0]	44.0 [28.0–70.0]	<0.0001
ALT (IU/mL)	38.0 [26.0–61.5]	44.0 [28.0–70.0]	35.0 [24.0–56.0]	<0.0001
γ-GT (IU/mL)	70.5 [38.0–175.0]	73.0 [42.0 -161.5]	67.0 [35.0–179.0]	0.485
ALP	95.0 [72.0–135.0]	88.0 [70.0–115.0]	100.0 [74.0–144.0]	<0.0001
Total bilirubin (mg/dL)	0.9 [0.6–1.4]	0.8 [0.6–1.2]	0.9 [0.6–1.7]	0.005
Albumin (g/dL)	4.2 [3.8–4.6]	4.4 [4.1–4.6]	4.1 [3.5–4.5]	<0.0001
Creatinine (mg/dL)	0.8 [0.7–1.0]	0.8 [0.7–1.0]	0.8 [0.7–1.0]	0.120
Platelet count (10³/µL)	191.0 [135.0–245.0]	205.0 [163.0–250.0]	173.0 [120.0–238.0]	<0.0001
Total cholesterol (mg/dL)	168.2 [129.5–210.9]	184.1 [148.5–218.2]	153.0 [119.0–198.0]	<0.0001
HDL (mg/dL)	42.0 [34.0–51.0]	41.0 [34.9–51.0]	43.0 [32.0–50.9]	0.482
LDL (mg/dL)	94.4 [67.0–127.1]	106.0 [81.8–134.6]	83.0 [62.0–113.0]	<0.0001
Triglyceride (mg/dL)	128.0 [87.0–188.7]	140.8 [99.2–230.2]	118.0 [83.5–170.5]	<0.0001
FIB-4 score, index	1.8 [1.0–3.5]	1.5 [0.9–2.4]	2.2 [1.1–4.5]	<0.0001
LSM by VCTE, kPa	9.6 [6.1–26.6]	8.1 [5.6–12.9]	13.5 [6.4–41.3]	<0.0001
FIB-4 categories, n (%)				<0.0001
<1.3	349 (39.1)	176 (47.7)	173 (33.0)	
1.3–2.67	237 (26.5)	118 (32.0)	119 (22.7)	
≥2.67	307 (34.4)	75 (20.3)	232 (44.3)	
LSM categories, n (%)				<0.0001
<8 kPa	338 (40.4)	163 (48.9)	175 (34.7)	
8–12 kPa	138 (16.5)	74 (22.2)	64 (12.7)	
≥12 kPa	361 (43.1)	96 (28.8)	265 (52.6)	
Advanced fibrosis (≥F3)^b^, n (%)	402 (45.0)	119 (32.2)	283 (54.0)	<0.0001

^a^ Comparisons between participants with MetALD and ALD were performed using the Student t test or the Wilcoxon rank-sum test for continuous variables, as appropriate, and the χ^2^ test or Fisher exact test for categorical variables.

^b^ Defined by a liver biopsy showing fibrosis stage 3–4 or liver stiffness measurement on vibration-controlled transient elastography ≥12 kPa.

Abbreviations: ALD, alcohol-associated liver disease; FIB-4, Fibrosis-4 index; HDL, High-density lipoprotein-cholesterol; LDL, low-density lipoprotein-cholesterol; LSM, liver stiffness measurement; MetALD, metabolic dysfunction and alcohol-associated liver disease; NH, non-Hispanic; VCTE, vibration-controlled transient elastography.

### Characteristics of biopsy-proven participants and differences with the VCTE-only group

The main differences in baseline clinical characteristics between biopsy-proven SLD subtypes and those evaluated exclusively with VCTE (without liver biopsy, as an explanatory analysis) are summarized in Supplemental Table S2, https://links.lww.com/HC9/C442. Compared with the biopsy group, participants in the VCTE-only group were more often men (81.6% vs. 74.5%; *p*=0.018), more frequently Hispanic (37.8% vs. 18.7%; *p*<0.0001), more likely to have a primary diagnosis of ALD (71.2% vs. 30.9%; *p*<0.0001), and less likely to have dyslipidemia (42.0% vs. 57.6%; *p*<0.0001). Median FIB-4 and LSM by VCTE were higher among individuals without liver biopsy (FIB-4: 1.9 vs. 1.6; *p*=0.009; LSM: 10.1 vs. 9.2 kPa; *p*=0.013), with a higher proportion of individuals in higher-risk FIB-4 and LSM categories (both *p*<0.001) (Supplemental Table S2, https://links.lww.com/HC9/C442).

Among biopsy-proven participants, 192 (69.1%) patients were diagnosed with MetALD and 86 (30.9%) with ALD. Overall, the median age was 53 years, and 74.5% were male. Type 2 diabetes and hypertension were present in 29.5% and 38.8%, respectively. The median FIB-4 index was 1.6 [1.0–2.6], and LSM was 9.2 kPa [6.5–15.1]. A total of 90 (32.4%) patients showed advanced fibrosis, and 47 (16.9%) participants had cirrhosis. When grouped by diagnosis, patients with ALD showed a higher prevalence of advanced fibrosis (53.5% vs. 22.9%) and cirrhosis (32.6% vs. 9.9%) in the group, compared with MetALD. Other variables, including sex, BMI, and some laboratory tests, were not significantly different between biopsy-proven MetALD and ALD (Supplemental Table S3, https://links.lww.com/HC9/C442).

### Performance of NITs for the detection of advanced fibrosis

**FIGURE 2:**
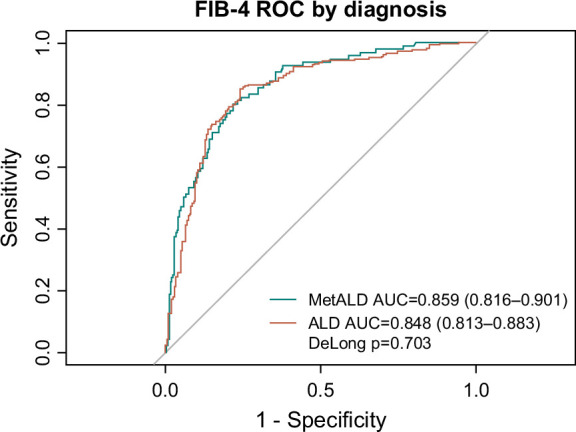
ROC curves comparing the diagnostic performance of FIB-4 for detecting advanced fibrosis in individuals with MetALD and ALD, using VCTE as the reference standard (overall cohort). AUC (95% CI) and DeLong *p* value are shown. Abbreviations: ALD, alcohol-associated liver disease; FIB-4, Fibrosis-4 index; MetALD, metabolic dysfunction and alcohol-associated liver disease; ROC, receiver operating characteristic; VCTE, vibration-controlled transient elastography.

**FIGURE 3:**
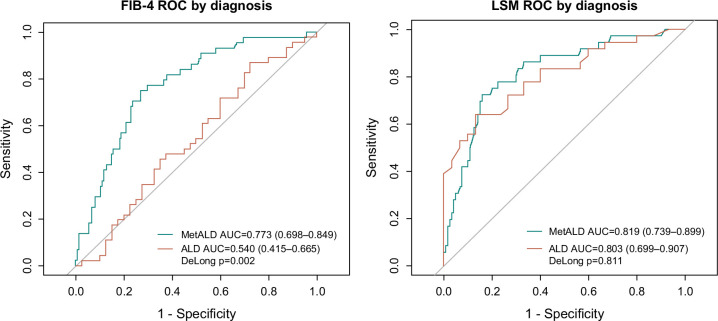
ROC curves comparing the diagnostic performance of FIB-4 and LSM in individuals with MetALD and ALD for detecting advanced fibrosis, using histology as the reference standard (biopsy-proven cohort). AUC (95% CI) and DeLong *p* values are shown. Abbreviations: ALD, alcohol-associated liver disease; FIB-4, Fibrosis-4 index; LSM, liver stiffness measurement; MetALD, metabolic dysfunction and alcohol-associated liver disease; ROC, receiver operating characteristic.

In the overall cohort, FIB-4 discriminated advanced fibrosis with an AUC of 0.857 (95% CI, 0.831–0.883). Using the rule-out threshold of 1.3, sensitivity was 92.8%, specificity 53.4%, PPV 60.1%, and NPV 90.7%. Using the rule-in threshold of 2.67, sensitivity was 66.6%, specificity 89.5%, PPV 82.8%, and NPV 77.9% (Table 2). Discrimination was comparable between diagnosis subtypes: AUC was 0.859 (95% CI, 0.816–0.901) in MetALD and 0.848 (95% CI, 0.813–0.883) in ALD, with no statistically significant difference (*p*=0.7032) (Supplemental Table S4, https://links.lww.com/HC9/C442 and Figure 2). When assessed among the biopsy-proven participants exclusively, FIB-4 showed an AUC of 0.72 (95% CI, 0.658–0.782) and LSM showed an AUC of 0.833 (95% CI, 0.775–0.890) for detecting advanced fibrosis (Table 3). When stratified by SLD classification, FIB-4 discriminated better in MetALD than ALD (AUC, 0.773 vs. 0.540, *p*=0.002). In contrast, LSM showed similar accuracy across groups (MetALD 0.819 vs. ALD 0.803, *p*=0.811) (Supplemental Table S5, https://links.lww.com/HC9/C442 and Figure 3). Distribution of valid biopsies and advanced fibrosis by country in SLD, and specifically in MetALD/ALD, is also provided. (Supplemental Table S6-S7, https://links.lww.com/HC9/C442).

**TABLE 2:** Overall diagnostic performance of FIB-4 for advanced fibrosis: AUC and operating characteristics at prespecified thresholds (all groups)

NIT	n	AUC (95% CI)	Cut point	Sensitivity (%)	Specificity (%)	PPV (%)	NPV (%)
FIB-4	837	0.857 (0.831–0.883)	1.3	92.8	53.4	60.1	90.7
						2.67	66.6	89.5	82.8	77.9

Abbreviations: FIB-4, Fibrosis-4 index; NIT, noninvasive test; NPV, negative predictive value; PPV, positive predictive value.

**TABLE 3:** Overall diagnostic performance of FIB-4 and liver stiffness measurement for advanced fibrosis: AUC and operating characteristics at prespecified thresholds (biopsy group)

NIT	n	AUC (95% CI)	Cut point	Sensitivity (%)	Specificity (%)	PPV (%)	NPV (%)
FIB-4	278	0.720 (0.658–0.782)	1.3	83.3	46.8	42.9	85.4
						2.67	38.9	82.4	51.5	73.8
LSM	222	0.833 (0.775–0.890)	8 kPa	90.3	53.3	48.1	92
						12 kPa	68.1	82.7	65.3	84.4

Abbreviations: FIB-4, Fibrosis-4 index; LSM, liver stiffness measurement; NIT, noninvasive test; NPV, negative predictive value; PPV, positive predictive value.

### Characterization and performance of NITs and clinical care pathways

**FIGURE 4:**
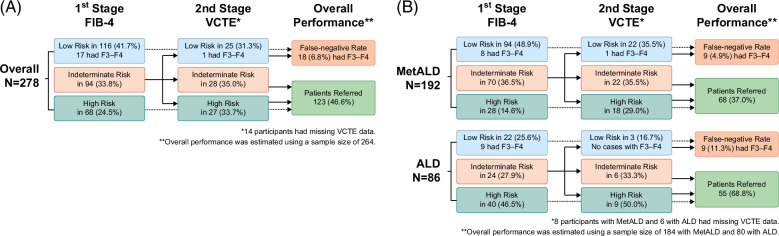
(A) Performance of the sequential pathway using FIB-4 and VCTE in individuals with biopsy-confirmed. (B) Performance of the sequential pathway using FIB-4 and VCTE in individuals with biopsy-confirmed, by diagnosis. Abbreviations: FIB-4, Fibrosis-4 index; VCTE, vibration-controlled transient elastography.

Using clinical care pathways with FIB-4 as a first step and VCTE as a second step in those with indeterminate FIB-4 among biopsy-proven participants, 116 (41.7%) patients were low risk by FIB-4, 94 (33.8%) were indeterminate, and 68 (24.5%) were high-risk. Of the 94 patients with indeterminate risk by FIB-4, 25 (31.3%) had low risk, while 28 (35.0%) and 27 (33.7%) patients had indeterminate and high risk, respectively, by VCTE. After the full 2-step algorithm, the false-negative rate among patients labeled low risk was 6.8%, 17 missed at FIB-4, and 1 missed at VCTE, while 123 (46.6%) should be referred for advanced care (Figure 4A).

In biopsy-proven MetALD, FIB-4 classified 94 (48.9%) as low, 70 (36.5%) as indeterminate, and 28 (14.6%) as high risk, with advanced fibrosis in 8 people in low risk. Among the FIB-4–indeterminate group, VCTE further split risk to 22 (35.5%) with a low, 22 (35.5%) indeterminate, and 18 (29.0%) high, showing risk stratification by FIB-4, and the second-stage VCTE showed broadly concordant enrichment for advanced fibrosis (Figure 4B). In ALD, the FIB-4 strata showed 22 (25.6%) with a low-risk FIB-4, while 24 (27.9%) were indeterminate, and 40 (46.5%) were high risk, whereas VCTE within the indeterminate stratum showed 3 (16.7%) with low-risk VCTE, 6 (33.3%) indeterminate, and 9 (50.0%) high risk (Figure 4B). Participants with MetALD showed a lower false-negative rate compared with ALD (4.9% vs. 11.3%) under the full 2-step pathway. The proportion of patients who should be referred to secondary care was 37.0% and 68.8% in the MetALD and ALD groups, respectively.

## DISCUSSION

In this retrospective, well-characterized cohort of participants with MetALD and ALD, we demonstrated that FIB-4 discriminated well in MetALD and non–biopsy-proven ALD, but was poor in patients with ALD undergoing liver biopsy, making it less reliable as a stand-alone triage method in ALD. Moreover, LSM by VCTE outperformed FIB-4 for detecting advanced fibrosis and remains consistent across MetALD and ALD. The 2-step clinical care pathway incorporating both NIT showed a good performance in detecting cases with advanced fibrosis in biopsy-proven MetALD, but its performance was substantially lower among biopsy-proven participants with ALD. Although our results support the use of clinical pathways using FIB-4 and VCTE in MetALD, they also underscore a major need for the refinement of screening methods in ALD.

In MetALD and ALD, commonly used NITs retain utility for ruling in or ruling out fibrosis, but the performance can be constrained by pathophysiological confounders introduced by metabolic dysfunction, alcohol use, or cholestasis. The FIB-4, aspartate aminotransferase to platelet ratio index (APRI) or nonalcoholic fatty liver disease fibrosis score, validated much in HIV/HCV coinfection,^19^^–^^21^ MASLD,^22^^–^^26^ and more recently explored in ALD,^27^^,^^28^ show acceptable degree of AUCs and high NPV for advanced fibrosis, but are vulnerable to high false positive rate in older patients (>65 years),^29^ as well as in those with higher levels of alcohol use, or in settings of thrombocytopenia unrelated to portal hypertension.^30^^–^^32^ In ALD, FIB-4 and other scores have modest performance for ruling in fibrosis, and guidelines therefore recommend their use primarily as a low-cost screening method. Alcohol use and associated systemic inflammation transiently increase AST, γ-GT, and LSM, so active drinking can lead to substantial overestimation of fibrosis on scores or LSM by VCTE.^33^^,^^34^ Similarly, cholestasis elevates bilirubin and γ-GT, and may inflate composite panels (eg, FibroTest, Fibrosure, Enhanced liver fibrosis), and obesity and metabolic inflammation also impair LSM by VCTE reliability and may require higher cutoffs.^35^^,^^36^ These biological features may partly explain the reduced diagnostic performance of FIB-4 observed in the biopsy-proven ALD subgroup in our study. In ALD, elevations in AST driven by ongoing alcohol exposure and thrombocytopenia related to mechanisms other than portal hypertension (such as alcohol-associated bone marrow suppression or nutritional deficiencies) may influence the components of FIB-4 independently of fibrosis stage. As a result, fibrosis risk-stratification strategies developed primarily in MASLD populations may not translate directly to ALD.

To address the limitations of single cutoffs and the variability of current NITs, clinical guidelines now favor a sequential strategy and dual thresholds (rule-out and rule-in) rather than a single decision point. Using a low cutoff with very high sensitivity minimizes false negatives for ruling out disease, while a high cutoff with very high specificity limits false positives for ruling in disease. In practice, this means starting with a broadly available lab-based score (eg, FIB-4), then moving to an imaging test (eg, VCTE) when results are indeterminate or above a referral threshold. In various previous studies, decent performance of the algorithms in clinical practice in patients with MASLD in different settings has been shown. Particularly in MetALD, scarce data on the identification of significant fibrosis exist, showing that clinical pathways are an acceptable method to stratify liver fibrosis.^37^ However, the sample size was very small, and the gold standard was defined by magnetic resonance elastography, not liver biopsy. Cost-effectiveness of the process in regard to health service utilization has also been demonstrated in multiple studies, showing that the 2-step approach is supported in effectively classifying individuals according to their risks by reducing unnecessary liver biopsies, thereby reducing health care costs in MASLD.^38^^–^^40^

This study has several strengths that reinforce the validity and clinical relevance of its findings. First, it represents one of the largest and most diverse cohorts of MetALD and ALD evaluated with standardized clinical algorithm pathways, leveraging data from 34 centers across 15 countries and thereby improving generalizability, which adopted the classification of the recent 2023 Delphi consensus. Second, our analysis is one of the few studies to assess the performance of clinical care algorithms to detect advanced fibrosis in individuals with MetALD and ALD. Finally, setting the biopsy-proven fibrosis as the gold standard and including LSM as an explanatory endpoint allowed this study to draw accurate and generalizable conclusions regarding the diagnostic performances of NITs.

Several limitations should also be acknowledged when interpreting these results. The cross-sectional design inherently precludes evaluation of longitudinal outcomes and dynamic changes in fibrosis. Selection bias is also likely, as included patients were drawn predominantly from hepatology and tertiary centers, and those who have VCTE or biopsy results (thus high prevalence of advanced fibrosis), and the distribution of MetALD and ALD differed substantially between the biopsy and VCTE-only groups, potentially influencing AUC estimates and limiting extrapolation to low-prevalence primary care settings. Specifically, PPVs and NPVs are primarily determined by the cohort prevalence and should not be generalized to lower-prevalent populations. In such settings, a greater proportion of patients would likely be classified as low risk, resulting in higher NPVs but lower PPVs. In addition, differences between the biopsy-confirmed and VCTE-only groups likely reflect real-world clinical decision-making, as the choice of fibrosis assessment modality is influenced by multiple factors, including diagnostic uncertainty, impact on clinical management, patient preference, and local availability of noninvasive testing.

Moreover, reliance on self-reported alcohol intake and the center-subjective assessment protocol of alcohol intake introduces a risk of misclassification of SLD diagnosis, particularly in individuals with fluctuating or underreported drinking patterns. Finally, in participants without biopsy, VCTE served as the reference standard for advanced fibrosis, which may inflate estimates of FIB-4 performance and does not fully address confounding from alcohol-specific laboratory results or cholestasis.

Also, not all centers adhered to a guideline-mandated sequential pathway, and biopsy decisions were often made independently of FIB-4/LSM. However, in this retrospective data set, we can still approximate reasonably how a sequential strategy would perform using available test pairs. Furthermore, the biopsy-proven ALD subgroup in this study was relatively small, which limits the precision of performance estimates and should be considered when interpreting these findings. Lastly, the multinational composition of this cohort, spanning 16 countries across Asia, the Americas, and Europe, is a strength but also a potential source of heterogeneity in NIT performance. In a prespecified subgroup analysis, the diagnostic pattern observed in the Americas was broadly consistent with the overall cohort, while the Asian subgroup showed a reversed pattern in which FIB-4 outperformed LSM, a finding that may reflect population-level differences in body composition, fibrosis etiology, or the applicability of Western-derived cutoff values. Given the limited sample size within each region, these findings should be interpreted cautiously, and future studies with larger region-specific cohorts are warranted to better characterize geographic and ethnic variability in NIT diagnostic accuracy.

Our findings have several implications for clinical practice, as we discussed earlier. First is the continuous use of LSM (VCTE) as one of the preferred NITs for detecting advanced fibrosis in individuals with MetALD and ALD, given its consistently higher accuracy and its relative robustness across the spectrum of the disease. Also, although FIB-4 appears adequate for initial risk stratification in MetALD, it is demonstrated that current thresholds and step-wise algorithms may require refinement, such as age-adjusted cutoffs or implications of dynamic drinking patterns, in an accurate manner. Looking ahead, there is a clear need for larger, prospective studies that track changes in dynamic alcohol use, metabolic alterations, and cholestasis over time, and that directly compare new biomarker panels and imaging methods in the SLD spectrum. We also need practical, risk-based care pathways that are adapted to different settings, such as primary care or hepatology clinics, and supported by implementation research, which will be essential to translate these findings into improved earlier intervention, timely referral, and ultimately better liver-related outcomes.

In conclusion, in this large, well-characterized cohort of MetALD and ALD, the use of clinical care pathways to stratify liver fibrosis risk using FIB-4 and LSM by VCTE showed a good performance in MetALD, supporting its wide use. However, this clinical pathway resulted in a clinically meaningful proportion of missed advanced fibrosis among patients with ALD, suggesting that new algorithms to stratify liver fibrosis are required in this setting. The issue of ongoing alcohol consumption may also be an important element to add to any new algorithm, on ALD, that may be facilitated with the implementation and dissemination of the new biomarkers, such as PeTH.

## Supplementary Material
