# Supplementary material for: Global multicenter validation of noninvasive fibrosis assessment pathways in MetALD and ALD
Source: Hepatol Commun. 2026 Jul 27;10(8):e01020. doi: 10.1097/HC9.0000000000001020 (PMC13412714; doi:10.1097/HC9.0000000000001020)

**APPENDIX:** Global Multicenter Validation of Noninvasive Fibrosis Care Pathways in MetALD and ALD

**Table of contents:**

| **Content** | **Page** |
| --- | --- |
| Supplementary Table 1 | 2 |
| Supplementary Table 2 | 3 |
| Supplementary Table 3 | 5 |
| Supplementary Table 4 | 7 |
| Supplementary Table 5 | 8 |
| Supplementary Table 6 | 10 |
| Supplementary Table 7 | 12 |
| Supplementary Table 8 | 14 |
| Supplementary Figure 1 | 15 |

**Supplementary Table 1.** Summary of the countries and the number of participants enrolled and included in the analysis with metabolic dysfunction and alcohol-associated liver disease (MetALD) and alcohol-associated liver disease (ALD). Data were collected across 35 centers in 16 countries during 2013–2025.

| **Continent** | **Countries** | **Number of centers included** | **Number of participants enrolled** | **Number of participants in the analysis** |
| --- | --- | --- | --- | --- |
| **The Americas** | Argentina | 4 | 52 | 33 |
|  | Brazil | 4 | 47 | 8 |
|  | Canada | 1 | 70 | 5 |
|  | Chile | 2 | 67 | 59 |
|  | Colombia | 2 | 23 | 9 |
|  | Dominican Republic | 1 | 24 | 4 |
|  | Mexico | 4 | 121 | 102 |
|  | Peru | 1 | 45 | 42 |
|  | United States | 4 | 233 | 30 |
|  | Venezuela | 1 | 1 | - |
| **Asia** | China | 2 | 17479 | 98 |
|  | India | 2 | 293 | 221 |
| **Africa** | Egypt | 1 | 2 | 2 |
| **Europe** | Portugal | 1 | 133 | 72 |
|  | Spain | 3 | 149 | 134 |
|  | United Kingdom | 2 | 77 | 74 |

**Supplementary Table 2.** Baseline characteristics of participants by fibrosis assessment

| **Characteristics** | **Only VCTE (N=615)** | **Liver biopsy (N=278)** | **p-value** |
| --- | --- | --- | --- |
| Age (years) | 51.0 [42.0 - 61.0] | 53.0 [44.0 - 62.0] | 0.154 |
| Male, n. (%) | 502 (81.6%) | 207 (74.5%) | 0.018 |
| Race/ethnicity, n. (%)  *NH White*  *NH Black/African American*  *Hispanic*  *Asian*  *Undisclosed or other* | 124 (20.2%)  3 (0.5%)  232 (37.8%)  246 (40.1%)  9 (1.5%) | 135 (48.6%)  1 (0.4%)  52 (18.7%)  85 (30.6%)  5 (1.8%) | <0.0001 |
| Body mass index (kg/m2) | 28.1 [24.4 - 31.6] | 28.0 [24.7 - 31.2] | 0.979 |
| Weight (kg) | 78.0 [68.0 - 90.0] | 79.8 [70.0 - 90.9] | 0.124 |
| Type 2 diabetes mellitus, n (%) | 160 (26.1%) | 82 (29.5%) | 0.337 |
| Hypertension, n (%) | 281 (45.9%) | 108 (38.8%) | 0.058 |
| Dyslipidemia, n (%) | 257 (42.0%) | 160 (57.6%) | <0.0001 |
| SLD Subtype  MetALD  ALD | 177 (28.8%)  438 (71.2%) | 192 (69.1%)  86 (30.9%) | <0.0001 |
| FIB-4 score, index | 1.9 [1.0 - 4.0] | 1.6 [1.0 - 2.6] | 0.009 |
| Liver stiffness measurement (LSM), kPa | 10.1 [6.0 - 36.0] | 9.2 [6.4 - 14.7] | 0.013 |
| FIB-4 categories, n (%)  < 1.3  1.3–2.67  ≥ 2.67 | 233 (37.9%)  143 (23.3%)  239 (38.9%) | 116 (41.7%)  94 (33.8%)  68 (24.5%) | <0.0001 |
| LSM categories, n (%)  < 8 kPa  8–12 kPa  ≥ 12 kPa | 251 (40.8%)  78 (12.7%)  286 (46.5%) | 87 (39.2%)  60 (27.0%)  75 (33.8%) | <0.0001 |
| Laboratory testing  *AST (IU/mL)*  *ALT (IU/mL)*  *𝛄-GT (IU/mL)*  *Alkaline phosphatase*  *Total bilirubin (mg/dL)*  *Albumin (g/dL)*  *Creatinine (mg/dL)*  *Platelet count (10³/µL)*  *Total cholesterol (mg/dL)*  *HDL (mg/dL)*  *LDL (mg/dL)*  *Triglyceride (mg/dL)* | 40.0 [28.0 - 64.0]  36.0 [25.0 - 55.0]  63.0 [34.0 - 147.5]  97.5 [73.0 - 140.0]  1.0 [0.6 - 1.7]  4.2 [3.6 - 4.6]  0.8 [0.7 - 1.0]  185.0 [127.0 - 244.0]  153.9 [121.2 - 198.0]  42.0 [33.0 - 50.6]  85.5 [63.9 - 117.5]  121.0 [82.0 - 173.0] | 41.0 [27.0 - 68.0]  46.0 [28.0 - 75.0]  85.0 [46.0 - 207.0]  90.0 [71.5 - 121.0]  0.8 [0.6 - 1.2]  4.3 [3.9 - 4.6]  0.8 [0.7 - 0.9]  204.0 [159.0 - 246.0]  188.0 [148.0 - 222.0]  43.0 [35.0 - 52.2]  107.9 [83.0 - 139.3]  142.5 [98.5 - 221.8] | 0.676  <0.0001  <0.0001  0.034  <0.0001  0.002  0.833  0.002  <0.0001  0.073  <0.0001  <0.0001 |

**Supplementary Table 3.** Baseline characteristics of the biopsy-proven cohort, by diagnosis

| **Characteristics** | **Global (N=278)** | **MetALD (N=192)** | **ALD (N=86)** | **p-value** |
| --- | --- | --- | --- | --- |
| Age (years) | 53.0 [44.0 - 62.0] | 52.0 [42.0 - 59.0] | 56.0 [49.0 - 64.0] | 0.001 |
| Male, n. (%) | 207 (74.5%) | 148 (77.1%) | 59 (68.6%) | 0.177 |
| Race/ethnicity, n. (%)  *NH White*  *NH Black/African American*  *Hispanic*  *Asian*  *Undisclosed or other* | 135 (48.6%)  1 (0.4%)  52 (18.7%)  85 (30.6%)  5 (1.8%) | 85 (44.3%)  0 ( 0.0%)  25 (13.0%)  80 (41.7%)  2 ( 1.0%) | 50 (58.1%)  1 ( 1.2%)  27 (31.4%)  5 ( 5.8%)  3 ( 3.5%) | <0.0001 |
| Body mass index (kg/m2) | 28.0 [24.7 - 31.2] | 27.5 [24.5 - 31.2] | 29.1 [26.3 - 31.6] | 0.127 |
| Weight (kg) | 79.8 [70.0 - 90.9] | 78.0 [69.9 - 90.0] | 82.0 [70.4 - 93.0] | 0.342 |
| Type 2 diabetes mellitus, n (%) | 82 (29.5%) | 61 (31.8%) | 21 (24.4%) | 0.271 |
| Hypertension, n (%) | 108 (38.8%) | 70 (36.5%) | 38 (44.2%) | 0.276 |
| Dyslipidemia, n (%) | 160 (57.6%) | 118 (61.5%) | 42 (48.8%) | 0.066 |
| FIB-4 score, index | 1.6 [1.0 - 2.6] | 1.5 [0.9 - 2.2] | 2.5 [1.3 - 4.5] | <0.0001 |
| Liver stiffness measurement (LSM), kPa | 9.2 [6.4 - 14.7] | 8.2 [6.0 - 11.8] | 14.1 [9.0 - 21.7] | <0.0001 |
| FIB-4 categories, n (%)  < 1.3  1.3–2.67  ≥ 2.67 | 116 (41.7%)  94 (33.8%)  68 (24.5%) | 94 (49.0%)  70 (36.5%)  28 (14.6%) | 22 (25.6%)  24 (27.9%)  40 (46.5%) | <0.0001 |
| LSM categories, n (%)  < 8 kPa  8–12 kPa  ≥ 12 kPa | 87 (39.2%)  60 (27.0%)  75 (33.8%) | 74 (47.4%)  45 (28.8%)  37 (23.7%) | 13 (19.7%)  15 (22.7%)  38 (57.6%) | <0.0001 |
| Laboratory testing  *AST (IU/mL)*  *ALT (IU/mL)*  *𝛄-GT (IU/mL)*  *Alkaline phosphatase*  *Total bilirubin (mg/dL)*  *Albumin (g/dL)*  *Creatinine (mg/dL)*  *Platelet count (10³/µL)*  *Total cholesterol (mg/dL)*  *HDL (mg/dL)*  *LDL (mg/dL)*  *Triglyceride (mg/dL)* | 41.0 [27.0 - 68.0]  46.0 [28.0 - 75.0]  85.0 [46.0 - 207.0]  90.0 [71.5 - 121.0]  0.8 [0.6 - 1.2]  4.3 [3.9 - 4.6]  0.8 [0.7 - 0.9]  204.0 [159.0 - 246.0]  188.0 [148.0 - 222.0]  43.0 [35.0 - 52.2]  107.9 [83.0 - 139.3]  142.5 [98.5 - 221.8] | 39.5 [27.0 - 59.0]  47.0 [29.0 - 77.0]  84.0 [47.5 - 184.5]  87.0 [70.0 - 107.0]  0.8 [0.5 - 1.1]  4.4 [4.1 - 4.7]  0.8 [0.7 - 0.9]  211.0 [170.5 - 250.0]  190.3 [156.5 - 221.8]  42.0 [35.4;52.8]  112.5 [85.5;139.0]  149.5 [102.0 - 241.7] | 49.0 [29.0 - 90.0]  41.5 [25.0 - 72.0]  100.0 [44.0 - 340.0]  102.0 [76.0 - 149.0]  0.8 [0.6 - 1.2]  4.1 [3.8 - 4.5]  0.8 [0.7 - 1.0]  175.5 [134.0 - 226.0]  178.0 [139.0 - 229.0]  43.0 [33.0 - 50.0]  100.0 [74.0 - 137.5]  129.0 [88.5 - 173.0] | 0.016  0.121  0.256  0.005  0.301  <0.0001  0.484  0.002  0.231  0.893  0.250  0.018 |
| Advanced fibrosis, n (%) | 90 (32.4%) | 44 (22.9%) | 46 (53.5%) | <0.0001 |
| Cirrhosis, n (%) | 47 (16.9%) | 19 (9.9%) | 28 (32.6%) | <0.0001 |

**Supplementary Table 4.** Diagnostic performance (AUC, 95% CI) of FIB-4 and LSM for detecting advanced fibrosis in MetALD and ALD, according to reference standard

| A. VCTE-referenced analysis (overall cohort) | | | | |
| --- | --- | --- | --- | --- |
|  | MetALD | ALD | | *p value** |
| FIB-4 | 0.859 (0.816 - 0.901) | 0.848 (0.813 - 0.883) | | 0.7032 |
| B. Pathology-proven analysis (biopsy cohort) | | | | |
|  | MetALD | ALD | | *p value** |
| FIB-4 | 0.773 (0.698 - 0.849) | 0.540 (0.415 - 0.665) | | 0.00208 (a) |
| LSM (VCTE) | 0.819 (0.739 - 0.899) | 0.803 (0.699 - 0.907) | | 0.811 (a) |
| C. Within-group comparison tests | | | | |
|  | MetALD | | ALD | |
| FIB-4 vs LSM | 0.160 (b) | | 0.00113 (b) | |

Values are expressed as **AUC (95% CI)**. AUCs were compared using the DeLong method.
p value: comparison between MetALD and ALD in the VCTE-referenced cohort.
(a) Between-group comparison (MetALD vs ALD).
(b) Within-group comparison (FIB-4 vs LSM).

**Supplementary Table 5.** Distribution of valid biopsies and advanced fibrosis by country in the full SLD cohort (n = 152,140)

| **Continent** | **Countries** | **Total observants (n)** | **Number of biopsies conducted, n (%)** | **Number of advanced fibrosis (stage 3-4), n (%)** |
| --- | --- | --- | --- | --- |
| **The Americas** | Argentina | 618 | 324 (52.3) | 102 (16.5) |
|  | Brazil | 1138 | 550 (48.3) | 166 (14.6) |
|  | Canada | 504 | 0 (0.0) | 0 (0.0) |
|  | Chile | 323 | 189 (58.5) | 27 (8.4) |
|  | Colombia | 296 | 136 (46.0) | 52 (17.6) |
|  | Dominican Republic | 156 | 2 (1.3) | 0 (0.0) |
|  | Mexico | 627 | 68 (10.9) | 17 (2.7) |
|  | Peru | 272 | 203 (74.6) | 42 (15.4) |
|  | United States | 313 | 47 (15.0) | 20 (6.4) |
|  | Venezuela | 64 | 0 (0.0) | 0 (0.0) |
| **Asia** | China | 144683 | 513 (0.3) | 133 (0.1) |
|  | India | 1207 | 101 (8.4) | 72 (6.0) |
|  | Singapore | 639 | 0 (0.0) | 0 (0.0) |
| **Africa** | Egypt | 175 | 175 (100.0) | 22 (12.6) |
| **Europe** | Portugal | 390 | 35 (9.0) | 14 (3.6) |
|  | Spain | 499 | 368 (73.8) | 124 (24.9) |
|  | United Kingdom | 127 | 81 (63.8) | 37 (29.1) |

**Supplementary Table 6**. Distribution of valid biopsies and advanced fibrosis by country in the MetALD/ALD cohort (n = 18,816)

| **Continent** | **Countries** | **Total observants (n)** | **Number of biopsies conducted, n (%)** | **Number of advanced fibrosis (stage 3-4), n (%)** |
| --- | --- | --- | --- | --- |
| **The Americas** | Argentina | 52 | 10 (19.2) | 6 (11.5) |
|  | Brazil | 47 | 6 (12.8) | 1 (2.1) |
|  | Canada | 70 | 0 (0.0) | 0 (0.0) |
|  | Chile | 67 | 2 (3.0) | 0 (0.0) |
|  | Colombia | 23 | 10 (43.5) | 4 (17.4) |
|  | Dominican Republic | 24 | 0 (0.0) | 0 (0.0) |
|  | Mexico | 121 | 21 (17.4) | 5 (4.1) |
|  | Peru | 45 | 0 (0.0) | 0 (0.0) |
|  | United States | 233 | 14 (6.0) | 8 (3.4) |
|  | Venezuela | 1 | 0 (0.0) | 0 (0.0) |
| **Asia** | China | 17479 | 5 (0.0) | 3 (0.0) |
|  | India | 293 | 4 (1.4) | 3 (1.0) |
| **Africa** | Egypt | 2 | 2 (100.0) | 1 (50.0) |
| **Europe** | Portugal | 133 | 7 (5.2) | 3 (2.7) |
|  | Spain | 149 | 101 (67.8) | 41 (27.5) |
|  | United Kingdom | 77 | 31 (40.3) | 15 (19.5) |

**Supplementary Table 7.** Baseline characteristics of the analysis cohort stratified by geographic region (Asia vs. Americas)

| **Characteristics** | **Asia (N = 319)** | **America (N = 262)** | **p-value** |
| --- | --- | --- | --- |
| Age (years) | 47.0 [40.0 - 55.0] | 52.5 [40.0 - 61.0] | 0.001 |
| Male, n. (%) | 312 (97.8%) | 176 (67.2%) | <0.0001 |
| Race/ethnicity, n. (%)  *NH White*  *NH Black/African American*  *Hispanic*  *Asian*  *Undisclosed or other* | 0 (0%)  0 (0%)  0 (0%)  319 (100%)  0 (0%) | 16 (6.1%)  0 (0.0%)  242 (92.7%)  1 (0.4%)  2 (32.8%) | 0.999 |
| Body mass index (kg/m2) | 26.2 [23.6 – 29.1] | 29.2 [25.5 – 32.4] | <0.0001 |
| Weight (kg) | 73.6 [66.2 - 82.0] | 80.0 [69.0 – 91.0] | <0.0001 |
| Type 2 diabetes mellitus, n (%) | 65 (20.4%) | 86 (32.8%) | <0.0001 |
| Hypertension, n (%) | 141 (44.2%) | 98 (37.4%) | 0.116 |
| Dyslipidemia, n (%) | 151 (47.3%) | 94 (35.9%) | <0.0001 |
| FIB-4 score, index | 2.8 [1.2 – 5.4] | 1.3 [0.8 - 2.4] | <0.0001 |
| Liver stiffness measurement (LSM), kPa | 28.6 [8.0 - 59.8] | 6.7 [4.9 – 10.3] | <0.0001 |
| FIB-4 categories, n (%)  < 1.3  1.3–2.67  ≥ 2.67 | 87 (27.3%)  67 (21.0%)  165 (51.7%) | 143 (54.6%)  57 (21.8%)  62 (23.7%) | <0.0001 |
| LSM categories, n (%)  < 8 kPa  8–12 kPa  ≥ 12 kPa | 74 (24.9%)  27 (9.1%)  196 (66.0%) | 152 (61.0%)  48 (19.3%)  49 (19.7%) | <0.0001 |
| Laboratory testing  *AST (IU/mL)*  *ALT (IU/mL)*  *𝛄-GT (IU/mL)*  *Alkaline phosphatase*  *Total bilirubin (mg/dL)*  *Albumin (g/dL)*  *Creatinine (mg/dL)*  *Platelet count (10³/µL)*  *Total cholesterol (mg/dL)*  *HDL (mg/dL)*  *LDL (mg/dL)*  *Triglyceride (mg/dL)* | 50.0 [33.0 – 77.8]  39.0 [27.1 – 62.5]  71.5 [42.0 – 164.0]  102.0 [77.5 - 141.5]  1.3 [0.8 – 2.4]  4.0 [3.4 - 4.5]  0.8 [0.7 – 0.9]  150.0 [100.5 - 213.5]  164.4 [129.0 - 204.0]  39.1 [34.0 - 47.6]  89.3 [69.1 - 119.8]  124.8 [81.4 – 204.2] | 32.0 [23.0 - 51.0]  34.0 [24.0 – 54.5]  49.0 [31.0 - 113.0]  84.0 [66.0 - 122.0]  0.7 [0.5 - 1.1]  4.3 [4.0 - 4.6]  0.8 [0.7 – 1.0]  221.0 [164.0 - 270.0]  163.0 [129.0 - 201.0]  43.0 [35.0 – 49.5]  93.5 [67.0 – 125.1]  133.0 [107.0 – 187.5] | <0.0001  0.011  <0.0001  0.034  <0.0001  <0.0001  <0.0001  <0.0001  0.556  0.106  0.499  0.073 |
| Advanced fibrosis (≥F3), n (%) | 202 (63.3%) | 59 (22.5%) | <0.0001 |

Supplementary Table 8. Diagnostic performance of FIB-4 and liver stiffness measurement for advanced fibrosis by geographic region, pathology-proven analysis

| NIT | n | AUC (95% CI) | Cut point | Sensitivity (%) | Specificity (%) | PPV (%) | NPV (%) |
| --- | --- | --- | --- | --- | --- | --- | --- |
| 1. Asia | | | | | | | |
| FIB-4 | 82 | **0.863** (0.750 - 0.977) | 1.3 | 80.0 | 69.4 | 26.7 | 96,2 |
|  |  |  | 2.67 | 40.0 | 94.4 | 50.0 | 91.9 |
| LSM | 60 | **0.747** (0.452 – 1.000) | 8 kPa | 66.7 | 74.1 | 22.2 | 95.2 |
|  |  |  | 12 kPa | 66.7 | 94.4 | 57.1 | 96.2 |
| 1. America | | | | | | | |
| FIB-4 | 48 | **0.531** (0.361 – 0.702) | 1.3 | 87.5 | 37.5 | 41.2 | 85.7 |
|  |  |  | 2.67 | 31.2 | 65.6 | 31.2 | 65.6 |
| LSM | 35 | **0.820** (0.661 – 0.978) | 8 kPa | 92.9 | 33.3 | 48.1 | 87.5 |
|  |  |  | 12 kPa | 42.9 | 95..2 | 85.7 | 71.4 |

Supplementary figure 1. Performance of the sequential FIB-4 and VCTE pathway in Asia and the Americas subgroups, pathology-proven analysis


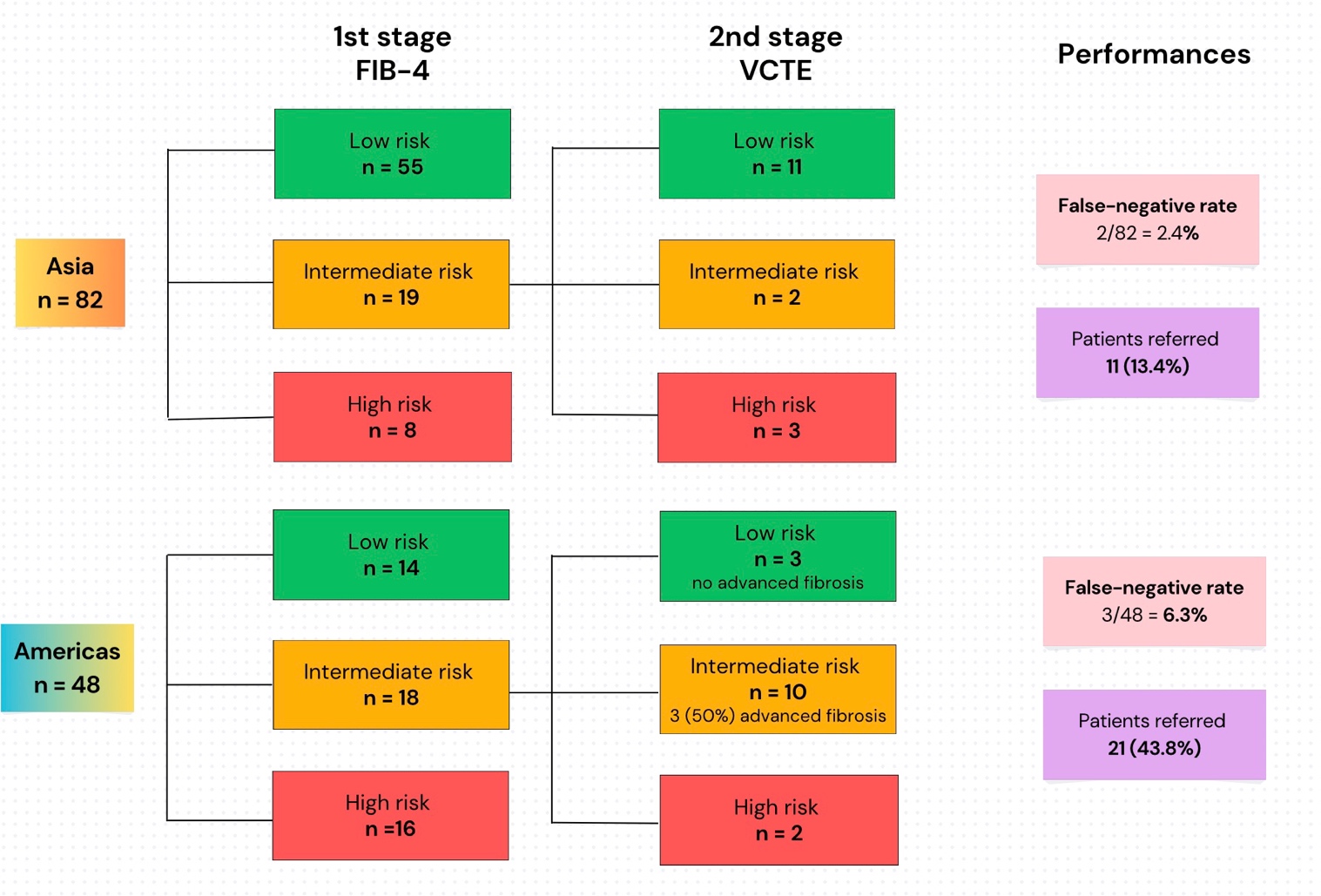

Supplement: Supplementary file 1 [file hc9-10-e01020-s001.docx]
